# Effects of Eurycoma longifolia Jack supplementation on eccentric leg press exercise-induced muscle damage in rugby players

**DOI:** 10.5114/biolsport.2023.119290

**Published:** 2022-09-22

**Authors:** Ahmad Zawawi Zakaria, Jad Adrian Washif, Boon Hooi Lim, Kazunori Nosaka

**Affiliations:** 1 Sports Performance Division, Institut Sukan Negara Malaysia (National Sports Institute of Malaysia), Malaysia; 2 Sports Centre, University of Malaya, Malaysia; 3 Centre for Human Performance, School of Medical and Health Sciences, Edith Cowan University, Australia

**Keywords:** Herb, Salivary testosterone, Delayed onset muscle soreness, Countermovement jump, Drop jump, Rugby 7s

## Abstract

*Eurycoma longifolia* Jack (ELJ) is a herbal plant that has androgenic and antioxidant effects. We investigated the short-term effect of ELJ supplementation on muscle damage induced by eccentric exercise. Eighteen young (19–25 years), well-trained rugby 7s players were assigned to an ELJ or a placebo (PLA) group (n = 9/group). Each participant took four 100-mg capsules a day for seven days prior to performing a leg press eccentric exercise to failure in a double-blind fashion. Peak force, peak power and jump height in countermovement jump (CMJ), drop jump reactive strength index (RSI), muscle soreness assessed by a 100-mm visual analogue scale, plasma creatine kinase (CK) activity, and salivary hormones were measured at 24 h before and 0.5, 24, 48, 72, and 96 h after the exercise. Changes in the variables over time were compared between the groups by two-factor mixed-design ANOVA. The number of eccentric contractions performed was similar (P = 0.984) between the ELJ (21 ± 5) and PLA groups (21 ± 5). Salivary testosterone and cortisol concentrations did not change (P > 0.05) after the supplementation for both groups. CMJ peak power (-9.4 ± 5.6%) and height (-10.6 ± 4.9%), and RSI (-15.2 ± 16.2%) decreased at 24 h after exercise (P < 0.05), and muscle soreness (peak: 89 ±10 mm) and plasma CK activity (peak: 739 ± 420 IU/L) increased after exercise (P < 0.05) without significant differences between groups. These results showed that 7-day ELJ supplementation prior to the leg press eccentric exercise had no significant effects on hormones, performance and muscle damage markers for the athletes.

## INTRODUCTION

*Eurycoma longifolia* Jack (ELJ), also known as Malaysian ginseng or tongkat ali, is a herbal species that has been used for medical purposes for thousands of years mainly in Southeast Asian countries. The root of ELJ contains several compounds (e.g., glycoprotein, eurycomanone) that are purported to be effective for anti-aging [1] and increasing androgen hormones such as testosterone [2, 3]. It was reported that consuming 200 mg/day of ELJ for one month increased serum testosterone concentration by 47% [2], and a dosage of 400 mg/day did not affect the urinary testosterone:epitestosterone ratio to the level that the World Anti-Doping Agency (WADA) considers as doping [3]. A dose of 600 mg/day ELJ ingestion was still found to be non-toxic [4]. However, potentially beneficial effects of ELJ on exercise are not well explored [3], although some studies have examined its effects on exercise performance [5, 6, 7]. Importantly, some athletes are taking an ELJ supplement in the belief that it improves performance. Thus, the effects of ELJ supplementation on athletic performance should be scrutinised.

Ooi et al. [8] found that supplementation of ELJ in the form of a drink containing 0.3 mg/kg body weight consumed every 20 min during a cycle ergometer trial until exhaustion did not change maximal oxygen consumption. Muhamad et al. [7] reported that a dosage of 150 mg/day ELJ for 7 days taken by recreational athletes did not show any beneficial effect on 20-min time trial running. However, ELJ supplementation that was consumed at a dosage of 100 mg/day for 5 weeks increased fat free mass by 4.1%, muscle strength of bench press by 6.8% and arm circumference by 5.8% in healthy adult men [5]. It appears that dosage and duration of ELJ supplementation are important factors when evaluating the ELJ ergogenic effects [3], and its effects on skeletal muscle are more promising than those on aerobic performance.

To the best of our knowledge, no previous study has investigated the effects of ELJ supplementation on exercise-induced muscle damage that is indicated by delayed onset muscle soreness (DOMS) and a prolonged loss of muscle function. Howatson et al. [9] investigated the anti-inflammatory and/or antioxidant effects of tart cherry juice. They reported that ingesting a commercially available tart cherry juice blend (473 ml) for 5 days before and for 2 days after a marathon run enhanced the recovery of muscle strength, and reduced increases in inflammation markers such as interleukin-6, C-reactive protein, and uric acid. Since ELJ contains antioxidants such as flavonoids [10], it is possible that ELJ supplementation affects the extent of muscle damage induced by eccentric exercise. Additionally, Talbott [11] reported that ELJ compounds (e.g., eurycomanone, flavonoids) improved hormonal profiles such as cortisol/testosterone ratio, mood, and wellbeing, while reducing stress and fatigue. Thus, it seems possible that ELJ compounds prevent overtraining syndrome, alleviate muscle soreness, and improve post-exercise recovery [11, 12]. However, these effects of ELJ have not been investigated.

Therefore, the present study examined the effects of ELJ supplementation for one week prior to exercise in comparison to the placebo (PLA) condition on changes in testosterone and some indirect markers of muscle damage after leg press eccentric exercise performed by elite athletes. We hypothesized that muscle function would recover faster and there would be less DOMS after exercise when ELJ was supplemented in comparison to the placebo condition.

## MATERIALS AND METHODS

### Participants

The present study recruited 18 elite male rugby 7s players who had represented Malaysia for major international tournaments such as the Commonwealth Games. Their mean ± SD (range) age, height, body mass, and competitive experience were 22 ± 2 (19–25) years, 1.73 ± 0.06 (1.63–1.85) m, 85 ± 10 (68–110) kg, and 5 ± 1 (3–7) years, respectively. The participants were ranked by the countermovement jump (CMJ) height, and assigned to one of the two groups, ELJ (n = 9) or PLA (n = 9), based on an ABBA procedure (block randomisation). In this format, a player with the best CMJ height was assigned to the ELJ group, players with the second and third best height to the PLA group, the fourth best player to the ELJ group, and so on. The inclusion criteria were male, international-level players, having at least three years of resistance training experience, and 100% commitment to the study including taking supplements as instructed, and reporting to a laboratory for all data collection sessions. Players with musculoskeletal injuries and/or illnesses in the previous month prior to data collection were excluded. Based on a study design of repeated measures, a priori sample size analysis using G*Power revealed that achieving an effect size of 0.50 (arbitrarily) with an alpha level of 0.05 and power of 0.90 would require a total sample size of 16.

Their physical characteristics shown above were not significantly (P > 0.05) different between groups. They were not allowed to use any supplements other than that provided to them during the study period. They rested for 48 hours prior to the first testing session, and were not allowed to engage in any physically demanding activities until the end of the experiment period (i.e., for 7 days before exercise and for 4 days after exercise). Specifically, no resistance training was conducted during this period; only light to moderate field training sessions (i.e., maintenance) focusing on tactical and strategy were performed by the players. Written informed consent was obtained from all participants, and this study was approved by the Institutional Research Committee (077–2018), in agreement with the Declaration of Helsinki.

### Study design

This double-blind, parallel design study was conducted at the National Sports Institute of Malaysia (Kuala Lumpur, Malaysia). The independent variable was the supplementation (ELJ vs PLA) and the dependent variables included the number of eccentric contractions performed in the leg press exercise to failure, peak force (PF), peak power (PP), and jump height in CMJ, reactive strength index (RSI) in drop jump (DJ), plasma creatine kinase (CK) activity, muscle soreness assessed by a visual analogue scale (VAS), and hormones in saliva (testosterone, cortisol). The eccentric exercise (leg press) was preceded by one-week daily supplementation of either ELJ or placebo (maltodextrin), and the dependent variables were measured at 24 hours before, 0.5, 24, 48, 72 and 96 hours after the exercise. The diet was standardised throughout the study period, and participants were instructed not to change their diet but to consume similar foods to what they ate prior to the study.

### Supplementation

The participants in the ELJ group ingested 4 capsules (1 capsule = 100 mg) of ELJ supplement, while the PLA group ingested the same amount of maltodextrin. The capsule was designed by the manufacturer (Biotropics Malaysia Berhad, Malaysia) to have the same shape, size, taste and colour; thus no one, including the investigators, knew which was which until the end of the study. All athletes were briefed regarding the study’s protocol including how the supplement should be consumed. The amount of the supplement was 400 mg per day for 7 days, and the participants took 200 mg (2 capsules) in the morning and 200 mg (2 capsules) in the afternoon with 250 ml of water for each occasion. This amount was chosen as it has been shown to increase testosterone and muscular strength [13], and has been demonstrated to be safe for long-term use [3].

### Exercise

All participants performed a bout of exercise consisting of repeated 5-s eccentric contractions of the knee extensors using a leg press machine with a 350-kg load to failure. For the eccentric phase (lowering the weight), the tempo was indicated to each participant by “verbal counting” along with a stopwatch. This phase was carried out in a controlled and balanced manner throughout the range of motion from a fully extended knee to a knee angle of ~90ᴼ. A brief pause of 1 second was inserted at the end of this movement before the weight was pushed back to the knee extended position by spotters; thus the participants performed the concentric phase without a load. One-repetition maximum (1-RM) strength of the leg press exercise was between 420 kg and 570 kg (average ± SD: 509 ± 53 kg) including the carriage’s mass of ~50 kg among the participants, which was determined prior to the study. Thus, the 350-kg weight was 61–83% (70 ± 8%) of the conventional 1-RM strength among the players. A repetition was deemed to be ‘failed’ if the lowering phase was less than 5 seconds, which was observed when each participant was no longer able to execute the next repetition. None of the participants had done the leg press eccentric exercise using this protocol prior to this study.

### Dependent variables

All dependent variables were measured at 24 hours prior to the exercise, and 24, 48, 72, and 96 hours after exercise. Additionally, CMJ and DJ measures were taken and muscle soreness assessment was performed at 30 minutes after exercise.

#### (a) Hormones

Saliva was collected to analyse testosterone and cortisol according to a standard procedure [12]. This required washing the mouth, resting and relaxing in a seated position for 10 min prior to the sample collection. One hour before the collection, the participants were instructed to avoid intake of any food or beverages including coffee. All samples were stored at -80°C for future analysis. The salivary testosterone and cortisol concentrations were determined using commercially available ELISA kits (Salimetrics LLC, Philadelphia, PA, USA). The assays were performed in duplicate, and the average value was used for further analysis. The intra-assay coefficient of variation was 8.4% and 10.0% for testosterone and cortisol, respectively.

#### (b) Peak force, peak power and height in CMJ, and reactive strength index in DJ

The lower limb muscle function was assessed by CMJ and DJ performed on a force platform (Fitness Technology, Adelaide, Australia), and the force data were sampled at 600 Hz. Each test procedure required each participant to perform four jumps with a 60-s rest between trials. During the CMJ, each participant placed his hands on his waist, and was instructed to jump as high as possible. Custom-designed software interfaced with the force plate provided PF, PP, and jump height. For the DJ, each participant was instructed to drop from a 30-cm box with his hands on the waist to eliminate arm swing, and to jump as high as possible after landing from the box by minimising the contact time. RSI was calculated by dividing the jump height by the ground contact time. For all tests, the highest value was used for further analysis [14, 15].

#### (c) Plasma CK activity

Blood (1 ml) was obtained by venepuncture from the antecubital vein, 30 μl of blood was pipetted onto a test strip, and CK activity was determined by an analyser (Reflotron Plus Chemistry Analyser, Roche Diagnostics, Indianapolis, IN, USA). The samples were analysed in duplicate and the average of the two measures was used for further analysis. The calculated inter-assay coefficient of variation was 6.4%.

#### (d) Muscle soreness

A VAS with a 100-mm line with “no pain” on one end (0) and “extremely painful” on the other end (100) was used to evaluate the extent of muscle soreness. This was assessed after stretching of the quadriceps, and each athlete was asked to indicate the level of pain on the scale.

### Statistical analyses

Shapiro-Wilk’s test of normality showed that each set of data was normally distributed (p > 0.05). The independent t-test was used to compare between groups for the number of eccentric contractions performed to failure. Two-factor mixed-design ANOVA was used to compare between the groups (ELJ, PLA) for changes in the dependent variables over time. Mauchly’s test of sphericity was used to check homogeneity of variance, and any violations of the assumption were corrected using the Greenhouse-Geisser or Huynh-Feldt adjustment. Additionally, Hedges’ *g* was calculated, and classified as trivial (g ≤ 0.20), small (0.20 < g ≤ 0.60), moderate (0.60 < g ≤ 1.20), large (1.20 < g ≤ 2.0), very large (2.0 < g ≤ 4.0), or extremely large (g > 4.0) [16]. All data were presented as mean ± standard deviation (SD). The accepted level of significance was set at p < 0.05. All data were analysed using IBM SPSS Statistics for Windows, version 26.0 (IBM Corp., Armonk, NY, USA).

## RESULTS

### Exercise

The number of eccentric contractions performed in the exercise to failure was similar between ELJ (21 ± 5) and PLA (21 ± 5) groups (p = 0.984).

### Hormones

No significant changes (p > 0.05) in testosterone, cortisol, and testosterone:cortisol ratio were found for both groups, and no significant interaction effect was evident (Table 1).

**TABLE 1:** Changes (mean ± SD) in salivary testosterone and cortisol concentrations and testosterone:cortisol ratio (T:C ratio) before (Pre), and 24, 48, 72 and 96 hours after eccentric leg press exercise for placebo (PLA) and *Eurycoma longifolia* Jack (ELJ) supplementation conditions.

		Pre	Post 24 h	Post 48 h	Post 72 h	Post 96 h
Testosterone (pmol/L)	PLA	505 ± 229	376 ± 208	478 ± 162	494 ± 214	440 ± 256
	ELJ	488 ± 313	414 ± 197	467 ± 210	570 ± 170	511 ± 222
Cortisol (nmol/L)	PLA	5.6 ± 3.1	6.1 ± 1.9	5.3 ± 1.8	6.0 ± 3.7	5.4 ± 2.7
	ELJ	5.6 ± 3.9	5.7 ± 3.1	5.7 ± 1.9	5.6 ± 1.9	5.7 ± 2.1
T:C Ratio	PLA	97 ± 33	78 ± 41	84 ± 35	107 ± 75	97 ± 64
	ELJ	103 ± 56	94 ± 65	83 ± 30	113 ± 55	105 ± 83

### Muscle damage markers

**FIG. 1:**
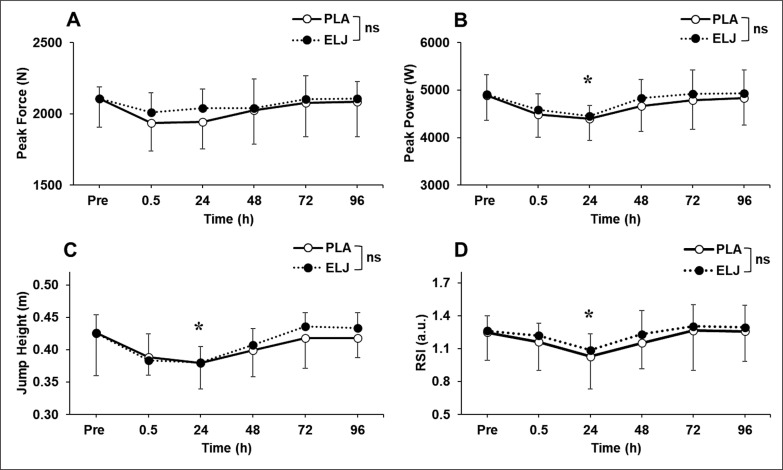
Changes (mean ± SD) in peak force (A), peak power (B) and jump height (C) during countermovement jump, and drop jump reactive strength index (D) before (Pre), and 0.5, 24, 48, 72 and 96 hours after eccentric leg press exercise for placebo (PLA) and *Eurycoma longifolia* Jack (ELJ) supplementation conditions. ns: not significantly different; *: significantly (P < 0.05) different from the Pre value.

Figure 1 shows changes in PF, PP and jump height in CMJ, and RSI from DJ over time. No significant differences (p > 0.05) were found for changes in any of the variables between groups. Compared to the baseline, significant (P < 0.01) decreases in PP (-9.4 ± 5.6%, *g* = -1.13, *moderate*), jump height (-10.6 ± 4.9%, *g* = -1.04, *moderate*), and RSI (-15.2 ± 16.2%, *g* = -0.88, *moderate*) were observed at 24 hours after exercise.

**FIG. 2:**
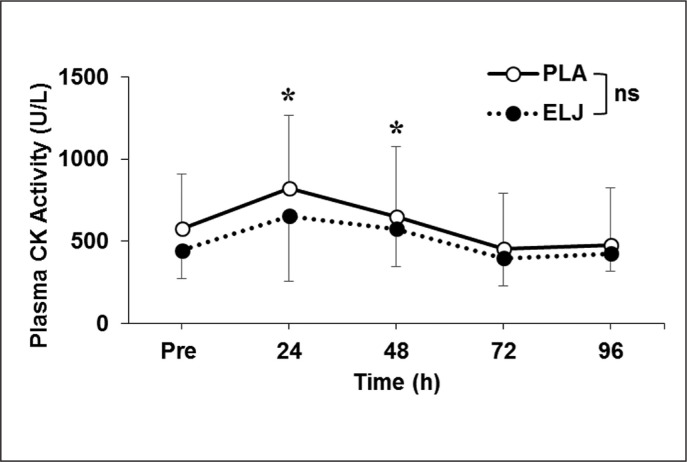
Changes (mean ± SD) in plasma creatine kinase (CK) activity before (Pre), and 24, 48, 72 and 96 hours after eccentric leg press exercise for placebo (PLA) and *Eurycoma longifolia* Jack (ELJ) supplementation conditions. ns: not significantly different; *: significantly (P < 0.05) different from the Pre value.

Plasma CK activity increased (P < 0.05) from the baseline (512 ± 265 IU/L) at 24 (49 ± 55%, *g* = 0.62, *moderate*) and 48 hours (21 ± 36%, *g* = 0.31, *small*) after exercise. However, no significant interaction effect (p > 0.05) was evident (Figure 2).

**FIG. 3:**
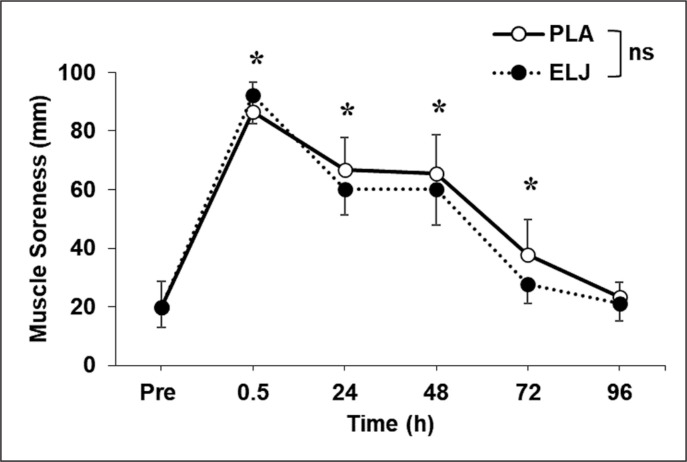
Changes (mean ± SD) in muscle soreness quantified by a 100-mm visual analogue scale before (Pre), and 24, 48, 72 and 96 hours after eccentric leg press exercise for placebo (PLA) and *Eurycoma longifolia* Jack (ELJ) supplementation conditions. ns: not significantly different; *: significantly (P < 0.05) different from the Pre value.

Muscle soreness increased (p < 0.05) at 0.5 (69 ± 12 mm, *g* = 7.35, *extremely large*), 24 (43 ± 14 mm, *g* = 4.50, *extremely large*) and 48 hours after exercise (43 ± 15 mm, *g* = 3.85, *extremely large*), and returned to the baseline by 72 hours after exercise (Figure 3). No significant (p > 0.05) difference was evident for the changes between groups.

## DISCUSSION

The results showed that the supplementation did not affect the salivary testosterone and cortisol concentrations (Table 1) nor the number of eccentric contractions performed to failure. Muscle damage was induced by the eccentric exercise indicated by significant decreases (*moderate* changes) in muscle function shown by the CMJ and DJ parameters at 24 hours after exercise, and increases in DOMS and plasma CK activity (*small* to *moderate* changes) as shown in Figures 1–3. However, the changes were not significantly different between the ELJ and placebo groups. These results did not support the hypotheses and suggest that the ELJ supplementation does not attenuate muscle damage.

Tambi et al. [2] found that serum testosterone concentration increased 47% when individuals (50 ± 10 years) with testosterone deficiency (late-onset hypogonadism) consumed 200 mg of ELJ for one month. However, the present study did not find any changes in salivary testosterone and cortisol concentrations after the 7-day ELJ supplementation or following the eccentric exercise (Table 1). This may be due to a shorter supplementation period than the previous study (one month vs one week) and/or the sensitivity of the measurement (blood vs saliva). It is also possible that the hormonal responses to the ELJ supplementation are affected by the age and athletic status of the participants. The age of the athletes in the present study (19 to 25 years) was concomitant with the peak production of testosterone [17]; thus it is possible that the ELJ supplementation did not increase the testosterone further due to a ceiling effect. Furthermore, the participants of the current study were athletes who were well trained and exposed to high-intensity training regularly, allowing them to recover quicker from the strenuous exercise [18]. With the same number of eccentric contractions performed in the exercise between the ELJ and placebo groups (~21 repetitions), similar levels of hormones after the exercise between groups were also anticipated. Talbott et al. [1] stated that ELJ was a ‘maintainer’ or ‘restorer’ of normal testosterone levels, rather than a testosterone ‘booster’; thus the ELJ supplementation effect on testosterone is likely more relevant to middle-age and older adults whose testosterone levels are lower.

Decreases in CMJ and DJ variables were observed following the leg press eccentric exercise, but they returned to the baseline values by 48 hours after exercise (Figure 1). A prolonged decrease in muscle function indicates muscle damage [19]; thus the decreases in the variables at 24 hours after exercise are likely to indicate muscle damage. However, the magnitude of muscle damage appeared to be minor, since the decreases after exercise were relatively small, and no longer seen at 48 hours after exercise. This may be due to the ‘single set’ eccentric protocol (even though it was performed ‘to failure’) that might not have been intensive enough for the ‘well-built’ athletes to get severe muscle damage. Another potential explanation for the minor muscle damage in the present study may be related to the muscle group used. Some studies have detected less muscle damage in leg muscles, especially for the knee extensors, when compared with arm muscles [20, 21, 22]. The quadriceps have a pennate architecture, presenting an angular orientation of fibres relative to the force-generating axis [23]. Pennate muscles (e.g., quadriceps) are characterised by large cross-sectional areas, providing lower specific tension (or mechanical strain) per muscle unit during a maximal voluntary contraction, as compared to fusiform muscles (e.g., elbow flexors), which are composed of fibres that extend parallel to the muscle’s force-generating axis [21].

It was expected that the recovery of muscle function would be enhanced by the anti-inflammatory and antioxidant effects of the ELJ supplementation. Howatson et al. [9] claimed that tart cherry juice, which is rich in antioxidants, enhanced recovery following marathon running due to increased total antioxidative capacity, and reduced inflammation and lipid peroxidation. A recent review concluded that acute administration of antioxidants immediately before or during an exercise session delays fatigue and reduces muscle damage and oxidative stress markers [24]. However, such findings were not confirmed in the present study. It is possible that because of the minor muscle damage, and the fact that the muscles recovered within 48 hours after exercise, any anti-inflammatory and antioxidant effects of the ELJ on muscle damage markers were not clearly detected.

Increased plasma CK activity resulted from plasma membrane disruption [25], which was evident at 24 and 48 hours after exercise similarly in both ELJ and placebo groups, but the magnitude of increase was small (Figure 2). As discussed above, this suggests that the magnitude of muscle fibre damage was relatively low. Hicks et al. [26] reported that pre-damage CK is < 150 IU/L for recreationally active males, while the current study obtained a baseline CK of ~500 IU/L, which was considered to be still a normal level for most athletes [27]. The relatively high baseline CK activity was probably due to the larger muscle mass and greater daily activities of the participants in the study. In a study among active individuals, Hicks et al. [26] reported that serum CK activity increased > 10 times at 96 hours following an eccentric knee extension protocol (6 sets × 12 repetitions at 60° · s^−1^). The magnitude of the increases in CK activity in the current study (~50% at 24 hours after exercise) indicate that muscle fibre damage was minor.

Muscle soreness developed at a similar extent in both ELJ and PLA groups, and subsided by 72 hours after exercise (Figure 3). It has been shown that muscle soreness is more associated with connective tissue (endomysium, perimysium, epimysium) damage and inflammation than muscle fibre damage [28]. Thus, the anti-inflammatory effect of the ELJ was expected to alleviate damage and inflammation of the connective tissue. Vitamin C antioxidant effects have been shown to attenuate muscle soreness when prescribed before and after eccentric exercise [29, 30]. A similar effect was observed when tart cherry antioxidant was taken before, during, and after a marathon run [9]. Thus, it is possible that continuous ELJ supplementation after the eccentric exercise would have attenuated DOMS. It is important to note that the magnitude of changes in the indirect markers of muscle damage varied. Thus, it is necessary to include both physiological and psychological markers when investigating the effect of exercises on muscle damage.

The current study had some limitations. First, the supplementation period was relatively short (one week) in comparison with previous studies in which the supplementation period was more than 4 weeks [5, 13]. This could have restricted potential positive results of the supplement. However, it should be noted that a short-term intervention is desirable for most athletes and coaches. We did not quantify dietary intake of our participants; however, they were training and living in the same national training centre, and were provided with a similar quality of meals across all athletes during the data collection sessions, although specific caloric intake may be different among individuals. As mentioned previously, the magnitude of muscle damage was minor in the present study, and therefore the potential effects of ELJ supplementation might have been masked. It is interesting to examine the effects of the ELJ supplementation on more severe muscle damage conditions. The present study did not include assessment of sleep quality [31], although all players were encouraged to preserve their night sleep duration and bed timing during the study period, and no sleep issue was reported by them. Finally, the present study used elite male athletes, so the results of the present study cannot be generalised to other populations such as female athletes, older adults, and sedentary individuals. Since it is known that the magnitude of muscle damage is greater for non-resistance-trained than resistance-trained individuals [32, 33], it is possible that the effects of ELJ supplementation on eccentric exercise-induced muscle damage are better clarified by using different populations. As a final note, many different supplements without strong scientific evidence are used by athletes; therefore it is important to scrutinise their effects and report the facts even if no positive effects are found. This will help the athletes and create a “healthy” environment in sports communities.

## CONCLUSIONS

In conclusion, ELJ supplementation (400 mg daily for seven days) had no effects on muscle functions, hormones, and muscle damage markers among elite rugby players. A longitudinal study to observe ergogenic effects of ELJ supplementation is warranted.

### Practical applications

– *Eurycoma longifolia* Jack (ELJ) supplementation has been reported to enhance testosterone level, but 400-mg supplementation for a week did not affect salivary testosterone and cortisol concentrations in young male athletes.
– The ELJ supplementation did not affect the number of eccentric contractions in a leg press exercise to failure performed by young male athletes.
– Muscle damage induced by the leg press eccentric exercise was not attenuated by the ELJ supplementation.

## Data Availability

Data associated with the study are available from the corresponding author upon reasonable request.
